# Targeting Mitochondrial ROS Production to Reverse the Epithelial-Mesenchymal Transition in Breast Cancer Cells

**DOI:** 10.3390/cimb44110359

**Published:** 2022-10-29

**Authors:** Elena Monti, Alessandro Mancini, Emanuela Marras, Marzia Bruna Gariboldi

**Affiliations:** 1 Department of Biotechnology and Life Sciences (DBSV), University of Insubria, Via J.H. Dunant 3, 21100 Varese, Italy; 2 Department of Translational Medical Sciences, University of Campania “Luigi Vanvitelli”, 80138 Naples, Italy; 3 BioUp Sagl, 6900 Lugano, Switzerland

**Keywords:** EMT, UQCRB, ROS, hypoxia, HIF-1α

## Abstract

Experimental evidence implicates reactive oxygen species (ROS) generation in the hypoxic stabilization of hypoxia-inducible factor (HIF)-1α and in the subsequent expression of promoters of tumor invasiveness and metastatic spread. However, the role played by mitochondrial ROS in hypoxia-induced Epithelial-Mesenchymal Transition (EMT) activation is still unclear. This study was aimed at testing the hypothesis that the inhibition of hypoxia-induced mitochondrial ROS production, mainly at the mitochondrial Complex III UQCRB site, could result in the reversion of EMT, in addition to decreased HIF-1α stabilization. The role of hypoxia-induced ROS increase in HIF-1α stabilization and the ability of antioxidants, some of which directly targeting mitochondrial Complex III, to block ROS production and HIF-1α stabilization and prevent changes in EMT markers were assessed by evaluating ROS, HIF-1α and EMT markers on breast cancer cells, following 48 h treatment with the antioxidants. The specific role of UQCRB in hypoxia-induced EMT was also evaluated by silencing its expression through RNA interference and by assessing the effects of its downregulation on ROS production, HIF-1α levels, and EMT markers. Our results confirm the pivotal role of UQCRB in hypoxic signaling inducing EMT. Thus, UQCRB might be a new therapeutic target for the development of drugs able to reverse EMT by blocking mitochondrial ROS production.

## 1. Introduction

The Epithelial-Mesenchymal Transition (EMT) is a biological process characterized by typical biochemical changes of epithelial cells that allow the acquisition of a mesenchymal phenotype and by the loss of cell-cell interactions and apicobasal polarity [1]. These changes lead to increased invasiveness, resistance to stress and to apoptotic cell death, and increased production of extracellular matrix proteins [1,2,3]. During the process, specific transcriptional modulators reduce the expression of the membrane glycoprotein E-cadherin, leading to the destabilization of adherent junctions and promoting cell mobility while increasing the synthesis of the mesenchymal protein vimentin, which modifies the composition of the intermediate filaments of the cytoskeleton, favoring invasiveness. The activation of the EMT program depends on the synergy between extracellular signals originating from the tumor microenvironment and the genomic and epigenomic structure of the cells. A number of extracellular ligands, including transforming growth factor β (TGF β), as well as microenvironmental cues, such as a reduction in oxygen partial pressure (frequently encountered in solid tumors), have been shown to activate and maintain EMT [2,3,4]. Under hypoxic conditions, EMT is induced as part of a pleiotropic adaptive response that is largely controlled by transcription factors belonging to the Hypoxia-Inducible Factor (HIF) family. HIFs are heterodimeric transcription factors consisting of a common β subunit that is constitutively present in cells, and of an inducible α subunit, which is specific for each family member. Under normoxic conditions, the latter is continuously synthesized and degraded following hydroxylation of two critical proline residues (P402 and P564) by specific prolyl hydroxylases (PHD1—3), which allows the interaction with the Von Hippel-Lindau (VHL) E3 ubiquitin ligase and subsequent protein degradation [5,6].

PHD activity is oxygen-dependent, and also requires Fe (II) and oxoglutarate as cofactors. Thus, under hypoxic conditions, the inducible α subunit is stabilized and the heterodimeric transcription factor accumulates in the nucleus, binds to specific sequences in gene promoters, and modifies the expression of several target genes, including those encoding for transcriptional modulators involved in EMT (Slug, Snail, Twist, Zeb) [7,8,9].

A number of studies published in the late 1990s/early 2000s have shown that intracellular generation of reactive oxygen species (ROS) is implicated in the hypoxic stabilization of HIF-1α, providing a possible mechanism through which ROS may promote tumor invasiveness and metastatic spread. These studies emphasize the role played by the mitochondrial electron transport chain, and more specifically of complex III, as a source for HIF-1α-stabilizing ROS during hypoxia [10,11,12,13]. Mitochondrial complex III, also called Ubiquinol-cytochrome c oxidoreductase or cytochrome bc1 complex, transfers electrons from ubiquinol to cytochrome c and couples this redox reaction to the translocation of protons from the matrix to the intermembrane space through a mechanism known as “Q cycle” [14,15,16,17,18]. During this cycle unstable ubisemiquinone intermediates are formed that can transfer their unpaired electron to oxygen, leading to superoxide formation; mitochondrial superoxide dismutase (SOD) then converts superoxide to H_2_O_2_, which inhibits PHD activity by direct attack and/or by decreasing Fe (II) availability, thus causing HIF-1α stabilization. Subsequent studies have established the ubisemiquinone formed at the Qo site of complex III as the crucial electron source for hypoxic ROS production. Accordingly, specific Qo inhibitors, such as stigmatellin, are able to counteract hypoxic ROS production and HIF-1α stabilization, as well as some HIF-1-dependent cellular responses such as VEGF release and angiogenesis [19]. In contrast, Qi site inhibitors, such as antimycin A, increase superoxide production by complex III and are therefore ineffective in preventing hypoxic adaptations [14,20].

Recently, the ubiquinol-cytochrome c reductase binding protein (UQCRB), a subunit of mitochondrial Complex III, has been found to play a pivotal role in hypoxic mitochondrial ROS generation, thereby emerging as an important modulator of tumor angiogenesis by the upregulation of both hypoxic signaling. Interestingly, the small molecule Terpestacin and its derivatives, which specifically bind UQCRB, inhibit the angiogenic response to pro-angiogenic stimuli, such as hypoxia [21,22,23], making UQCRB a potential new therapeutic target for antiangiogenic drug development.

While the role of mitochondrial ROS in hypoxia-induced angiogenesis has been successfully established, their implication in EMT activation is still unclear. The present study was aimed at testing the hypothesis that the inhibition of hypoxia-induced mitochondrial ROS production could result in the reversion of EMT, in addition to decreased HIF-1α stabilization, thus providing a rationale for the development of drugs able to counteract tumor progression induced by the EMT by blocking mitochondrial ROS production. We first evaluated the role of hypoxia-induced ROS increase in HIF-1α stabilization, in a triple-negative breast cancer cell line that has often been used in the study of EMT, namely MDA-MB468. Secondly, we assessed the ability of a number of antioxidants, mostly targeting mitochondrial Complex III and including Terpestacin, to block ROS production and HIF-1α stabilization and prevent changes in EMT markers.

Finally, the specific role of UQCRB in hypoxia-induced EMT was evaluated by silencing its expression through RNA interference and assessing the effects of its downregulation on ROS production, HIF-1α levels and EMT markers.

## 2. Materials and Methods

### 2.1. Chemicals

Myxothiazol (Myxo), mito-TEMPO (mitoTP), standard chemicals, and cell culture reagents were purchased from Euroclone s.r.l. (Milan, Italy), unless otherwise indicated. Terpestacin was kindly provided by Dr. HoJeong Kwon (Yonsei University, South Korea).

### 2.2. Cell Lines, Drug Treatment and Hypoxia Induction

The breast cancer cell lines MCF7, MDA-MB468, and MDA-MB231 were originally obtained from American Type Culture Collection (Rockville, MD, USA) and recently authenticated by morphological inspection, growth curve analysis, and short tandem repeat profiling. MCF7 cells are hormone responsive, whereas the other two cell lines derive from triple negative breast carcinomas (TNBCs), a particularly aggressive and untreatable subset of breast cancers [24]. The 293FT cell line (Invitrogen, Milan, Italy) was used to produce replication-incompetent lentiviral particles. Cells were maintained in RPMI1640 (MCF7 and MDA-MB231; ECM2001L) and DMEM (MDA-MB468 and 293FT; ECB7501L) medium supplemented with 10% fetal bovine serum, 1% glutamine (ECB3000D), 1% antibiotic mixture, 1% sodium pyruvate (S8636) and 1% non-essential amino acids (ECB3054D) and incubated at 37 °C in a humidified 5% CO_2_ atmosphere. Cells were cultured for less than 10 weeks (18–20 passages) and were regularly checked for Mycoplasma (Molecular Biology Reagent Set *Mycoplasma* species, Euroclone, UK).

For all experiments, unless otherwise indicated, cells were seeded, treated 24 h later with Myxo (T5580, 1 μM), mitoTP (SML0737, 100 and 200 μM) or Terpestacin (25 μM) in serum-free medium and incubated for 48 h at 37 °C in normoxia (O_2_ 21%) or hypoxia (O_2_ 1%). Drug concentrations were based on literature data [22,25,26,27]. Hypoxia was induced by placing the cells for the indicated times inside a modular incubator chamber (Billups Rothenberg Inc., Del Mar, CA, USA) flushed with a mixture of 1% O_2_, 5% CO_2,_ and 94% N_2_ at 37 °C.

### 2.3. Scratch Wound Healing Assay

Cell migration was evaluated on the studied cell line by the scratch wound healing assay. Briefly, 7 × 10^5^ cells/well were grown (approximately to confluence) in 6-well plates, and a scratch was produced in the cell monolayers using a 100-μL pipette tip. The culture medium was then replaced by serum-free medium and the plates were incubated under normoxic or hypoxic conditions. Pictures of the scratch wound were taken immediately following scratch formation (0) and after 48 h incubation, through a camera connected to an Olympus IX81 microscope. The percentages of the open scratch wound were evaluated by the TScratch software.

### 2.4. Evaluation of ROS Levels

Intracellular ROS generation was evaluated using 2,7-dichlorodihydrofluorescein diacetate (DCFH-DA, D6883) as a probe. At the end of 48 h treatment with mitoTP (100 and 200 μM), Myxo (1 μM), or Ter (25 μM), under both normoxic or hypoxic conditions, the cells were detached, washed, and re-suspended (10^6^ cells/mL) in PBS containing 10 μM DCFH-DA; after 45 min incubation in the dark at 37 °C, cell samples were then analyzed with a FACSCalibur flow cytometer (Becton Dickinson Mountain View, CA, USA) and data were processed using CellQuestPro software (Becton Dickinson- version 6.0). DCFH-DA could not be used to analyze ROS production in infected cells as they also express GFP (Green Fluorescent Protein, see below); thus, for these experiments, DCFH-DA was replaced by dydroethidine (D7008, HE) [28]. Briefly, cells were detached, washed and resuspended (10^6^ cells/mL) in HE (25 μM in PBS); following 30′ incubation at 37 °C in the dark, samples were analyzed as described [28,29]. Intracellular ROS generation was quantitated in arbitrary units based on the mean fluorescence intensity (MFI).

### 2.5. Western Blot Analysis

Western blot analysis was carried out to detect the expression of HIF-1α, E-cadherin, N-cadherin, vimentin, cytokeratin-19, Snail, Slug, and UQCRB in whole cell lysates, following normoxic or hypoxic incubation and/or drug treatment. For whole lysates, cells were lysed in a buffer containing NaCl 120 mM, NaF 25 mM, EDTA 5 mM, EGTA 6 mM, sodium pyrophosphate 25 mM in TBS 20 mM pH 7.4, PMSF 2 mM, Na_3_VO_4_ 1 mM, phenylarsine oxide 1 mM, 1% (*v*/*v*) NP-40 and 10% Protease Inhibitor Cocktail. Protein concentration was determined by the BCA assay (23235, Thermo Fisher, Monza (MI), Italy); 100 μg of protein per sample was loaded onto polyacrylamide gels (8% or 12%) and separated under denaturing conditions. The protein bands were then transferred onto Hybond-P membranes (GE10600023, Sigma Aldrich, Milan, Italy) and Western blot analysis was performed by standard techniques, with mouse monoclonal anti-human HIF-1α (610958, BD Bioscience), E-cadherin (5085) and N-cadherin (13A9, Thermo Fisher, Monza (MI), Italy) antibodies; mouse monoclonal anti-human cytokeratin 19 (A53B, Thermo Fisher, Monza (MI), Italy) antibody; rabbit polyclonal anti-human vimentin, Snail and Slug antibodies (ABCAM). Equal loading of the samples was verified by re-probing the blots with an anti-mouse-β-tubulin antibody (SC-5274, Santa Cruz Biotechnology, Segrate (MI), Italy). Protein bands were visualized using a peroxidase-conjugated anti-mouse secondary antibody and the Westar Supernova Substrate (XLS-3, Cyanagen, Bologna, Italy).

### 2.6. Construction of Lentiviral Vectors

Lentiviral particles were generated using a second-generation transient expression system, composed of (i) the pCMV R8.74 packaging construct, (ii) the pMD2.G envelope expression construct, and (iii) the pLVTHM/GFP transfer vector, for silencing of UQCRB expression by RNA interference. The pLVTHM/GFP contains a green fluorescent protein (GFP) cDNA under the transcriptional control of an intronless human elongation factor 1-α (EF1-α-short) promoter. All constructs were kindly provided by Dr. Didier Trono (School of Life Sciences, Swiss Institute of Technology, Lausanne, Switzerland; www.epfl.ch/labs/tronolab/ (accessed on 25 May 2005)). The transfer vector pLVTHM/shUQCRB/GFP or pLVTHM/shScrambledUQCRB/GFP was generated as follows. A sense strand of 19 nucleotides specific for UQCRB, preceded by overhangs specific for MluI cloning, was designed to be followed by a short loop sequence (TTCAAGAGA), by the reverse complement of the sense strand, with five terminal thymidines to act as a RNA polymerase III transcriptional stop signal, and by a sticky sequence specific for ClaI. The forward oligonucleotide (5′-GCGTCCCCGACAGGATGTTTCGCATTATTCAAGAGATAATGCGAAACATCCTGTCTTTTTGGAAAT-3′ corresponding to the 300–319 nucleotides of UQCRB (NCBI Reference Sequence: NM_001199975.3), was annealed with a complementary reverse oligonucleotide (3′-AGGGGCTGTCCTACAAAGCGTAATAAGTTCTCTATTACGCTTTGTAGGACAGAAAAACCTTTAGC-5′) in annealing buffer (100 mM potassium acetate, 30 mM HEPES pH7.4, 2 mM magnesium acetate). Annealed oligos were phosphorylated with T4 Polynucleotide Kinase (New England Biolabs Ltd.) and cloned into the MluI-ClaI sites of a pLVTHM lentiviral vector. The transfer vector pLVTHM/shScrambledUQCRB/GFP was constructed in the same way (5′-GCGTCCCCAGTGTTGGACGATTATCACTTCAAGAGAGTGATAATCGTCCAACACTTTTTTGGAAAT-3′ sequence obtained from www.genscript.com (accessed on 15 January 2016)).

### 2.7. Generation of Lentiviral Particles and Target Cell Infection

Lentiviral particles pseudotyped through the VSV envelope glycoprotein were produced by co-transfecting 5 × 10^6^ 293FT cells with 40 µg of total plasmid DNA: the (i) pCMVDR8.74, (ii) pMD2.G, and (iii) pLVTHM/shUQCRB/GFP or pLVTHM/scrambledUQCRB/GFP vectors, with the calcium phosphate precipitation method, as previously described [30]. Transduction experiments were performed in a medium containing 4µg/mL polybrene (Sigma-Aldrich, Milan, Italy). Viral titration was performed by flow cytometer-counting GFP-expressing NIH3T3 cells 48 h after infection. For in vitro shRNA-UQCRB silencing, 60% confluent MDA-MB468 cells were infected for 4 h with 10 MOI lentiviral vectors; the particle-containing medium was then replaced with fresh medium and the cells were incubated at 37 °C for 48 h.

### 2.8. Evaluation of Cell Viability

Cell viability was evaluated through the dye exclusion assay following treatment and/or under normoxic and hypoxic conditions. Briefly, 2 *×* 10^5^ cells were seeded onto 6-well plates and 24 h later were treated and incubated for 48 h in the presence of 21% or 1% O_2_. Cells were then detached and counted using a Burker hemocytometer, following Trypan blue staining.

### 2.9. Statistical Analysis

Statistical analysis of the data obtained from flow cytometric studies, densitometric analysis of western blot results, and Scratch Wound Healing assay were performed by means of one- or two-way ANOVA, with Bonferroni’s test for multiple comparisons, using GraphPad PRISMsoftware (version 4.03).

## 3. Results

### 3.1. Characterization of Breast Cancer Cell Lines

**Figure 1:**
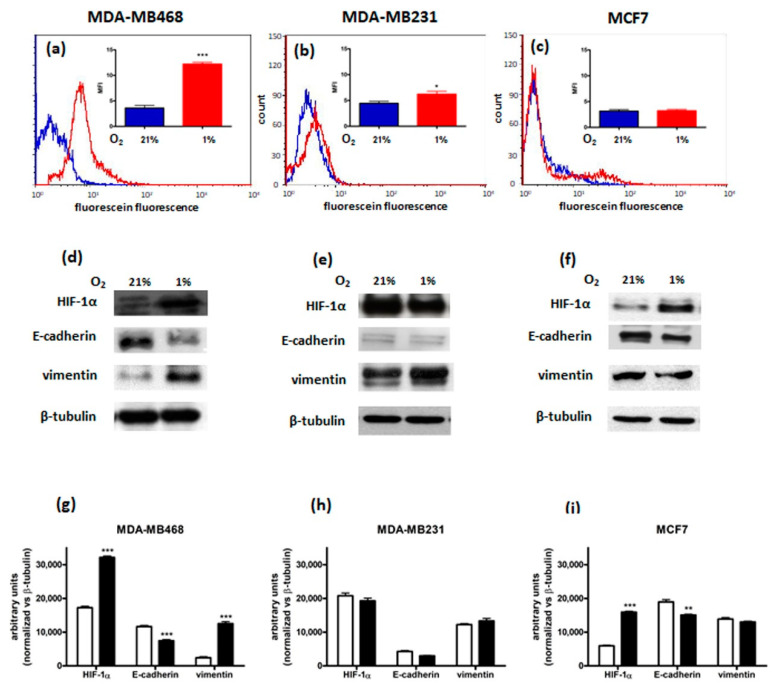
MDA−MB468, MDA−MB231 and MCF7 cells response to 48 h hypoxia (O_2_ 1%). (**a**–**c**): ROS levels (blue: O_2_ 21%; red: O_2_ 1%). Fluorescence intensity was quantitated based on the Median Fluorescence Intensity (MFI) and results obtained in 3 independent experiments are reported in the graph (*** *p* < 0.001 and * *p* < 0.05 vs. normoxia). (**d**–**f**): HIF−1α, E−cadherin and vimentin protein levels in MDA−MB468 (**d**), MDA-MB231 (**e**), and MCF7 (**f**) cell lines (representative western blot analysis out of 3 independent experiments with similar results). Densitometric analysis (**g**–**i**) were performed on all western blot experiments (*** *p* < 0.001 and ** *p* < 0.01 vs. normoxia).

Three breast cancer cell lines were initially considered for the present study, namely MCF-7, MDA-MB468, and MDA-MB231, representing three distinct subtypes of the disease [24,31]. MCF7 cells belong to the luminal A subtype, expressing hormone receptors and exhibiting the least aggressive behavior of all breast cancer subtypes. In contrast, both MDA-MB468 and MDA-MB231 derive from triple-negative breast cancers, lacking ER-α, PR, and HER-2 expression, and they belong to the basal A and basal B groups, respectively, based on GE profiling. To find the ideal breast cancer cellular model to test the hypothesis that the inhibition of hypoxia-induced mitochondrial ROS production could result in the reversion of EMT, in addition to decreased HIF-1α stabilization, the three cell lines were characterized concerning their migratory capacity and ability to increase ROS and HIF-1α protein levels and to exhibit changes in EMT markers in response to hypoxia. For this purpose, ROS, HIF-1α, E-cadherin, and vimentin levels and cell migration were evaluated following 48 h incubation of the cells under normoxic (O_2_ 21%) or hypoxic (O_2_ 1%) conditions. Figure 1 shows that in MDA-MB468 cells 48 h hypoxia-induced ROS levels increase, as indicated by a shift to the right on the fluorescein peak. Fluorescence intensity was quantitated in arbitrary units based on Median Fluorescence Intensity (MFI—Figure 1a). Differences in HIF-1α, E-cadherin, and vimentin expression were also observed following 48 h incubation in hypoxia. In particular, the E-cadherin-vimentin switch, typical of the EMT, was present, along with the expected increase in HIF-1α levels (Figure 1d). Moreover, this cell line showed intrinsic migratory capacity, both in normoxia and hypoxia, as indicated in Table 1, reporting the percentages of the open scratch wound.

A different pattern was observed in the other triple-negative breast cancer cell line characterized, namely MDA-MB231. In this cell line, under hypoxic conditions, migratory capacity and only a slight increase in ROS levels were observed (Table 1 and Figure 1b, respectively). However, the same EMT pattern was present both under normoxic and hypoxic conditions (Figure 1e), thus making MDA-MB231 cells unsuitable for our purposes. In contrast, MCF7 cells, representing a less aggressive breast cancer subtype (i.e., responsive to estrogens and progesterone), did not show either migratory activity or significant alterations in both ROS production and EMT-related protein levels following exposure to hypoxia (Table 1 and Figure 1c,f, respectively).

Therefore, only MDA-MB468 cells responded to hypoxic stimuli by increasing ROS and HIF-1α levels, as well as by activating EMT, as indicated by the decrease in E-cadherin and the increase in vimentin levels, and cell motility, in agreement with previously reported data [32,33,34]. In MDA-MB468 cells, hypoxia also induced an increase of the two E-cadherin transcription repressors, Snail and Slug (Figure S1), confirming the switch from epithelial to mesenchymal phenotype as reported by other authors [35]. However, the results obtained in this first part of the study suggest that such responses are highly cell/tumor type-dependent.

Based on these results, MDA-MB468 were chosen for further investigations.

### 3.2. Effects of the Inhibition of ROS Production on Hypoxia-Induced Responses

To confirm the role of hypoxia-induced ROS increase in EMT induction, the effects of the inhibition of ROS generation in hypoxic MDA-MB468 cells were evaluated following treatment with antioxidants/ROS-scavengers or inhibitors of the mitochondrial chain Complex III. In particular, the effects of Mito-Tempo (MitoTP), a mitochondria-specific antioxidant, were evaluated. Furthermore, the effects of two specific inhibitors of mitochondrial chain Complex III, the antibiotic Myxothiazol (Myxo), which targets the mitochondrial complex III at the Qo site, close to the heme group b566 [20,36] and Terpestacin (Ter), which inhibits the Mitochondrial Complex III by specifically binding the UQCRB (ubiquinol-cytochrome c reductase binding protein) subunit [37] were also evaluated.

#### 3.2.1. Decrease of ROS Levels Using MitoTP

**Figure 2:**
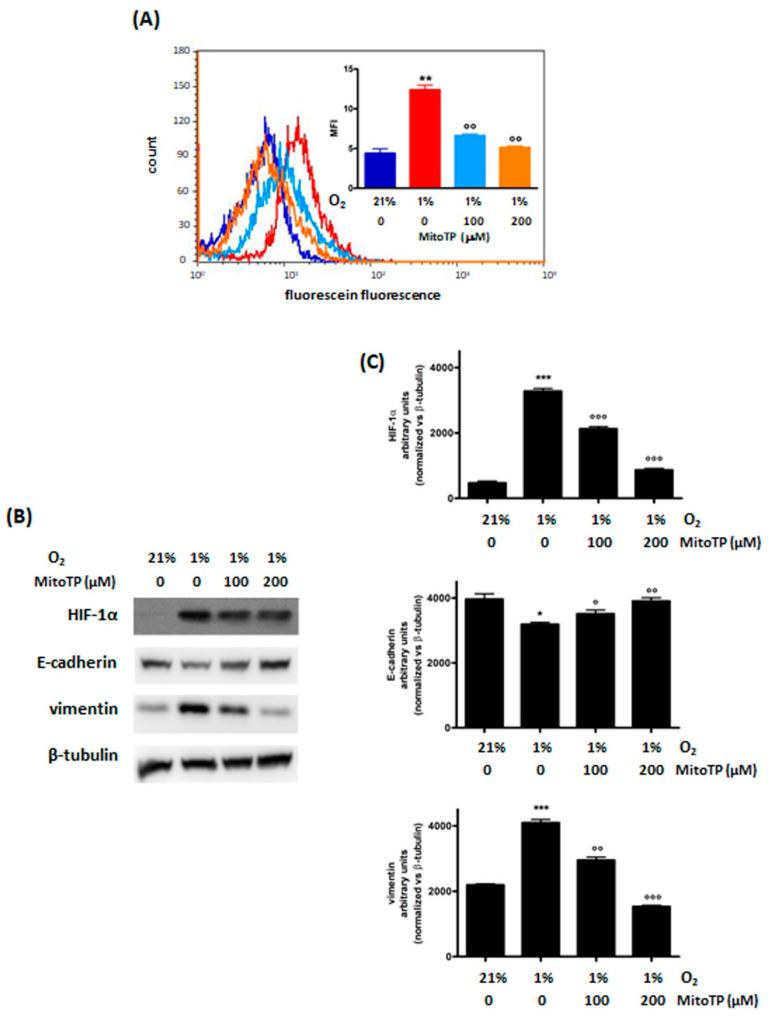
Effects of 48 h treatment with MitoTP (100 and 200 μM and incubation in normoxic (O_2_ 21%) or hypoxic (O_2_ 1%) conditions in MDA−MB468 cells: ROS (**A**) and HIF−1α, E−cadherin and vimentin (**B**) levels. The figure shows a representative flow cytometric and Western blot analysis out of 3 independent experiments with similar results. For flow cytometric analysis of ROS levels (blue line: normoxic control; red line: hypoxic control; light blue line: hypoxic MitoTP 100 μM treated cells; orange line: hypoxic MitoTP 200 μM treated cells) fluorescence intensity was quantitated based on the Median Fluorescence Intensity (MFI) and results obtained in 3 independent experiments are reported in the graph. Densitometric analysis (**C**) was performed on all experiments (* *p* < 0.05, ** *p* < 0.01 and *** *p* < 0.001 vs. normoxic control; ° *p* < 0.05, °° *p* < 0.01, °°° *p* < 0.001 vs. hypoxic control).

MitoTP is a small molecule that acts specifically as a mitochondrial ROS scavenger and was shown to inhibit cell migration inhibitor [38,39]. In our model, MitoTP significantly reduced ROS levels, in a dose-dependent manner (Figure 2A), and induced a parallel decrease in HIF-1α protein levels in hypoxic MDA-MB468 cells. These effects were associated with a decrease in vimentin and with an increase in E-cadherin levels (Figure 2B,C). Furthermore, MitoTP showed the same effect on cell survival in both normoxia and hypoxia (Figure S2).

#### 3.2.2. Decrease of ROS Levels Using Inhibitors of Mitochondrial Complex III

**Figure 3:**
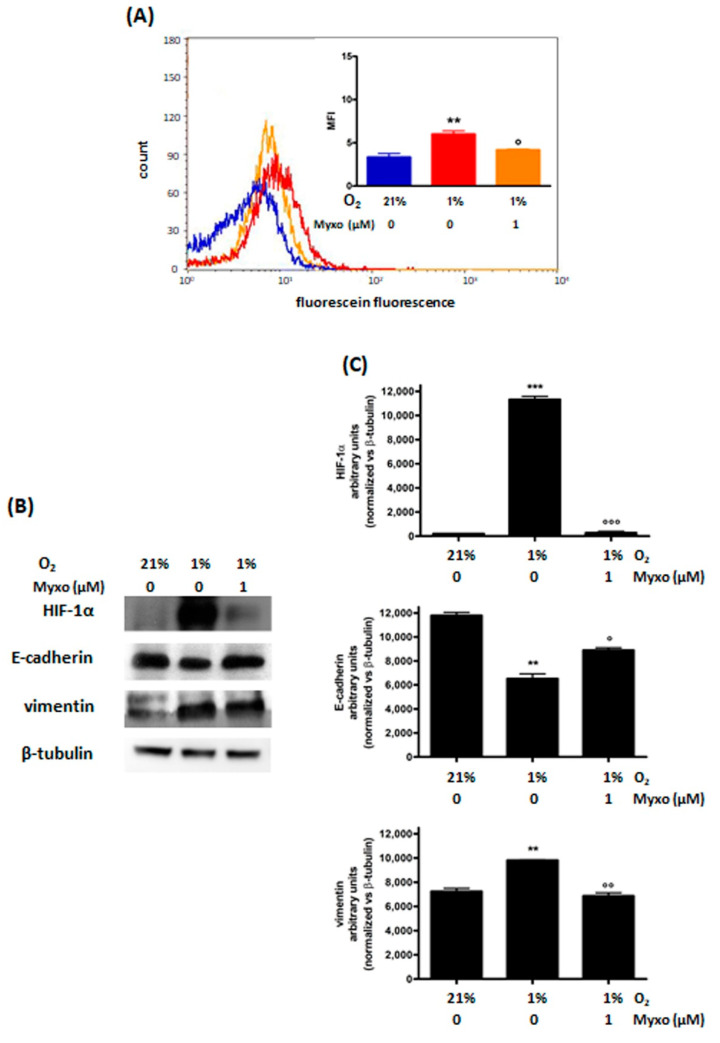
Effects of 48 h treatment with Myxo treatment (1 μM) and incubation in normoxic (O_2_ 21%) or hypoxic (O_2_ 1%) conditions in MDA−MB468 cells: ROS (**A**) and HIF−1α, E−cadherin and vimentin (**B**) levels. The figure shows representative flow cytometric and Western blot analyses out of 3 independent experiments with similar results. For flow cytometric analysis of ROS levels (**A**); blue line: normoxic control; red line: hypoxic control; orange line: hypoxic Myxo treated cells). The fluorescence intensity was quantitated based on the Median Fluorescence Intensity (MFI) and results obtained in 3 independent experiments are reported in the graph. Densitometric analysis (**C**) was performed on all experiments (*** *p* < 0.001 and ** *p* < 0.01 vs. normoxic control; °°° *p* < 0.001, °° *p* < 0.01 and ° *p* < 0.05 vs. hypoxic control).

Myxothiazol (Myxo) interacts with the Qo site, blocking electron transfer within complex III, thereby preventing ROS production, and inhibiting HIF-1α stabilization and HIF transcriptional activity in hypoxia. In agreement with these reports, we observed a significant decrease in hypoxia-induced ROS production following 48 h treatment with Myxo, as shown in Figure 3A. In MDA-MB468 cells Myxo was also able to significantly decrease hypoxia-induced HIF-1α and vimentin protein levels and increase hypoxia-decreased E-cadherin levels (Figure 3B,C). Furthermore, Myxo was shown to reduce the increase of Slug, N-cadherin, and Snail protein levels induced by 48 h incubation of the cells in hypoxia (Figure S3). A reduction in cell viability was observed following 48 h treatment; however, the same extent of viability reduction was induced by Myxo also in hypoxia (Figure S4).

**Figure 4:**
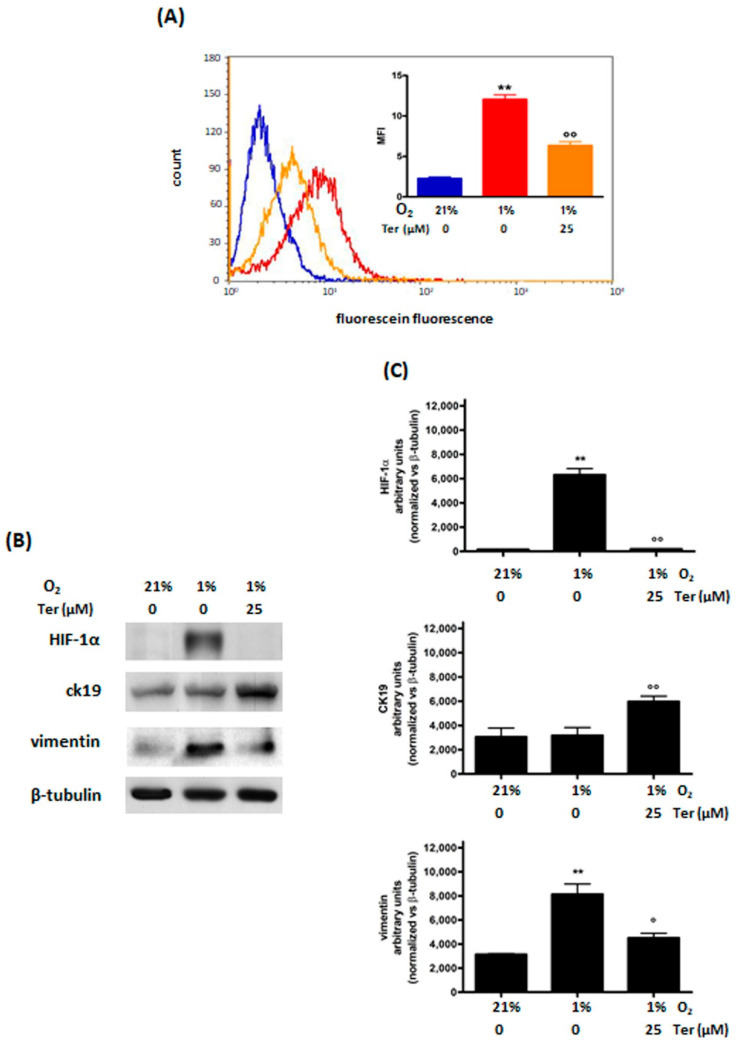
Effects of 48 h of treatment with Ter (25 μM) and incubation in normoxia (O_2_ 21%) or hypoxia (O_2_ 1%) in MDA−MB468 cells: ROS (**A**) and HIF−1α, cytokeratin 19, and vimentin (**B**) levels. The figure shows a representative flow cytometric and Western blot analysis out of 3 independent experiments with similar results. For flow cytometric analysis of ROS levels (**A**); blue: normoxic control; red: hypoxic control; orange: hypoxic Ter treated cells), fluorescence intensity was quantitated based on the Median Fluorescence Intensity (MFI), and results obtained in 3 independent experiments are reported in the graph. Densitometric analysis (**C**) was performed on all experiments (** *p* < 0.01 vs. normoxic control; °° *p* < 0.01 and ° *p* < 0.05 vs. hypoxic control).

In addition, in MDA-MB468 cells Ter significantly reduced the hypoxia-induced ROS production (Figure 4A). Furthermore, Ter was able to prevent hypoxia-induced HIF-1α stabilization, resulting in the reversion of the mesenchymal phenotype. For this set of experiments, CK19 was used as an epithelial marker instead of E-cadherin; Ter was found to prevent both the decrease in CK19 and the increase in vimentin levels induced by hypoxia (Figure 4B,C). Furthermore, treatment with Ter only produced a modest decrease in viability in normoxic MDA-MB468 cells, which is also observed under hypoxic conditions (Figure S5).

### 3.3. Effects of the UQCRB Silencing

Finally, the effects of UQCRB downregulation on ROS production and HIF-1α and EMT protein levels were evaluated on MDA-MB468/pLVTHM, MDA-MB468/scrambledUQCRB and MDA-MB468/shUQCRB, obtained by infection of MDA-MB468 cells, as indicated in the Materials and Methods section, following 48 h incubation in the presence of 21% or 1% O_2_. The effects of the UQCRB silencing on cell migration and cell viability were also evaluated.

**Figure 5:**
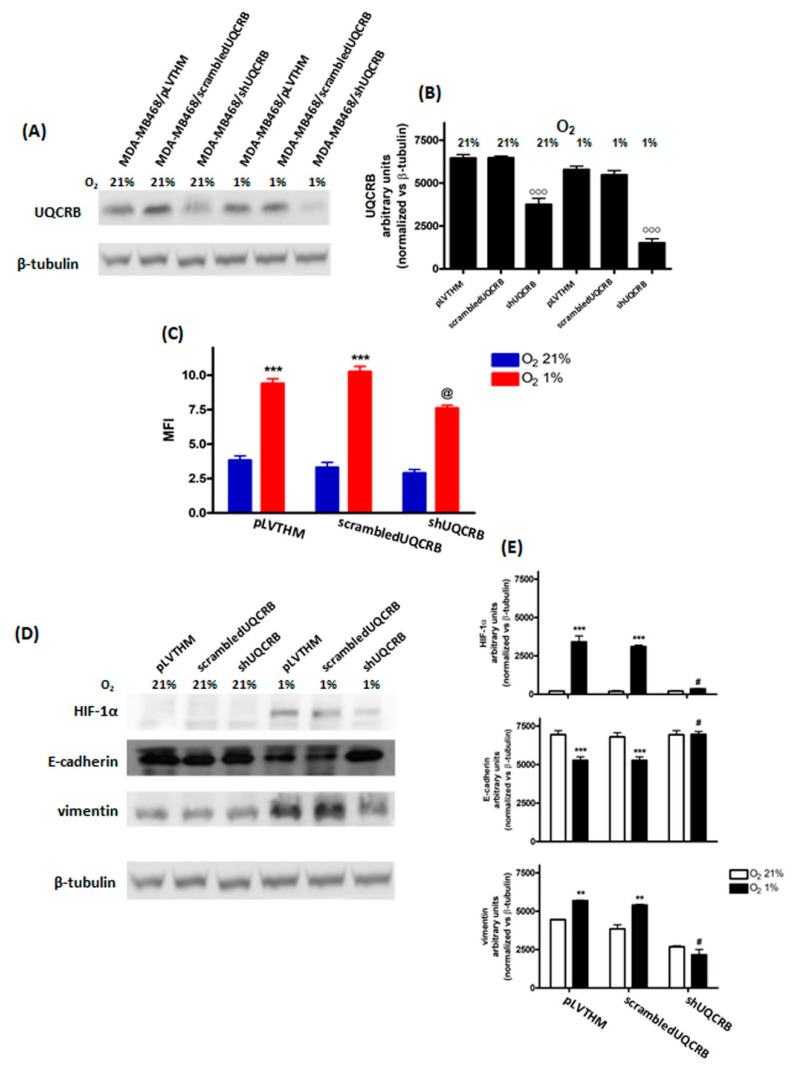
Effects of UQCRB silencing in MDA−MB468 cells, following 48 h incubation in normoxia (O_2_ 21%) or hypoxia (O_2_ 1%): UQCRB protein levels (**A**) and related densitometric analysis (**B**), ROS (**C**) and HIF-1α, E−cadherin and vimentin levels (**D**) and related densitometric analysis (**E**). The figure shows densitometric analysis of 3 independent western blot experiments. For flow cytometric analysis of ROS levels (**C**), fluorescence data are expressed as Median Fluorescence Intensity (MFI, blue: O_2_ 21%; red: O_2_ 1%). (°°° *p* < 0.001, *** *p* < 0.001 and ** *p* < 0.01 vs. normoxic control; @ *p* < 0.01 vs. hypoxic (O_2_ 1%) PLVTHMand scrambled; # *p* < 0.01 vs. hypoxic pLVTHMand scrambled).

The results from the western blot analysis of whole cell lysates showed that following the infection of MDA-MB468 cells with shUQCRB, the UQCRB protein levels were significantly reduced in both normoxic and hypoxic MDA-MB468/shUQCRB cells, indicating successful silencing of the protein (Figure 5A,B). As hypothesized, a significant reduction in hypoxia-induced ROS increase was observed in MDA-MB468/shUQCRB cells, compared to MDA-MB468/pLVTHM and MDA-MB468/scrambledUQCRB cell lines (Figure 5C and Figure S6).

UQCRB silencing abrogated HIF-1α stabilization and reversed both the decrease in E-cadherin and the increase in vimentin levels induced by hypoxia (Figure 5D,E). Snail levels were also decreased in hypoxic MDA-MB468/shUQCRB cells, compared to the two control cell lines (Figure S7).

**Figure 6:**
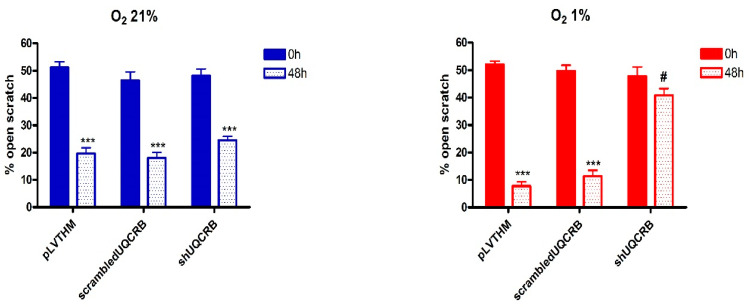
Percentage of open scratch wound in MDA-MB468/pLVTHM, MDA-MB468/scrambledUQCRB and MDA468/shUQCRB cells following 48 h incubation under normoxic (O_2_ 21%) and hypoxic conditions (O_2_ 1%) (mean S.E. of 3 independent experiments; two-ways ANOVA results: *** *p* < 0.001 vs. 0 h same condition; # *p*< 0.01 vs. MDA-MB468/pLVTHM and MDA-MB468/scrambledUQCRB 48 h).

**Figure 7:**
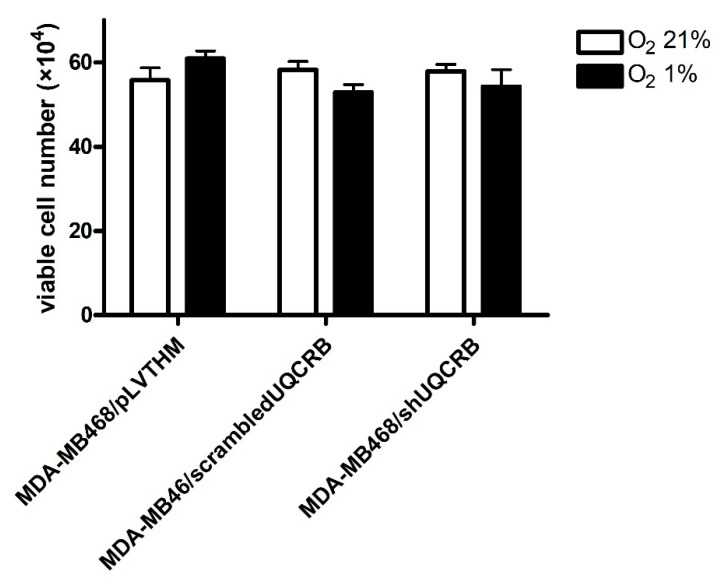
Effects of UQCRB silencing on MDA-MB468/pLVTHM, MDA-MB468/scrambledUQCRB, and MDA-MB468/shUQCRB cell viability in normoxia (O_2_ 21%) or hypoxia (O_2_ 1%) (mean ± S.E. of 3 independent experiments).

The results from the Scratch Wound Healing and viability assays are reported in Figure 6 and Figure 7, respectively, while representative images of the scratches are reported in Figure S8. Interestingly, UQCRB silencing results in a significant reduction of the migratory capability of MDA-MB468 cells mainly under hypoxic conditions, while in normoxia, only a modest decrease in migration was observed.

However, the viability of UQCRB-silenced cells was comparable to that of the two control cell lines, confirming that UQCRB inhibition does not significantly affect cell viability.

## 4. Discussion

The role of ROS in the activation of EMT, through HIF1α stabilization, is still unclear, despite the involvement of ROS and HIF-1α in this process which has been highlighted by a number of authors [40,41,42]. This evidence was also supported by data showing that antioxidants, such as SkQR1, NAC, Mito-CP, and Mito-TEMPO, are able to modulate EMT [41,43,44].

Recently, it has been demonstrated that hypoxia causes superoxide production by respiratory chain Complex III; the superoxide anion is then converted to H_2_O_2_, which inhibits PHD enzyme activity by direct attack and/or reduction of Fe (II) availability, causing HIF1α stabilization [27]. Thus, the hypoxic response, mediated by HIF-1α, modifies the expression of many target genes linked to EMT, including those encoding for the transcriptional modulators Snail, Slug, Twist, and Zeb [7,8,9].

Here, we have confirmed that the inhibition of hypoxia-induced mitochondrial ROS production through treatment with antioxidants leads to decreased HIF-1α stabilization and reverts the changes in some hypoxia-induced EMT markers in the TNBC cell line MDA-MB468. However, the aspecific effects of these antioxidants on both mitochondrial Complexes I and III also resulted in a cell viability reduction on cell viability. The novelty of the present work lies in the hypothesis that specific inhibition of hypoxia-induced mitochondrial ROS at the UQCRB site of the mitochondria Complex III, obtained both through Ter treatment or UQCRB silencing, results in the reversion of EMT, in addition to decreased HIF-1α stabilization and without significantly affecting cell viability.

To account for the discrepancies observed between this cell line and the other TNBC cell line characterized, namely MDA-MB231, it should be emphasized that TBNCs are far from homogeneous. While cell lines derived from this type of breast cancer are generally defined as basal-like, a first major distinction can be made based on gene clustering between the basal A group, displaying epithelial characteristics and often associated with *BRCA1* gene signatures, and the Basal B group, displaying mesenchymal and stem/progenitor-like characteristics. A more refined analysis of gene expression profiles has led to the classification of TNBCs into six different subtypes [45]. According to this classification, MDA-MB468 cells belong to the basal-like BL-1 subtype, displaying epithelial characteristics, whereas MDA-MB231 are assigned to the mesenchymal/stem-like subtype, which may well account for their more invasive behavior and for their pattern of expression of epithelial and mesenchymal markers even under normoxic conditions. Important differences have been detected in the genomic profiles of the two cell lines, including the presence of the activating KRAS^G13D^ mutation in MDA-MB-231 (which per se might confer a more aggressive behavior) [46]. In addition, this latter cell line has been shown to constitutively express the urokinase receptor uPAR, and silencing this receptor has been shown to lead to a more epithelial phenotype; in contrast, MDAMB468 cells express low uPAR levels in normoxia, but under hypoxic conditions, they upregulate its expression in a HIF-1-dependent fashion, and uPAR overexpression has been shown to reversibly induce EMT in this cell line [47]. These features may help explain the differences observed between the two cell lines in the preliminary phase of the present study.

Results obtained with the mitochondrial ROS scavenger MitoTP indicated that in MDA-MB468 cells reduction of ROS levels lead to the reduction of HIF-1α cellular levels and consequently to the mesenchymal-epithelial switch (i.e., increase in E-cadherin and decrease in vimentin protein levels), confirming the interplay between ROS increase, HIF-1α stabilization, and EMT induction.

Mitochondria are essential to hypoxia-induced ROS increase [10]. Specifically, complexes I and III of the mitochondrial electron transport chain represent the main sites of ROS production; however, recently Chandel demonstrated, then it was confirmed by others, that Complex III is the site responsible for the hypoxic production of ROS [15,48]. Using the so-called Q cycle, Complex III transfers electrons from ubiquinol to cytochrome c and contributes to the generation of an electrochemical proton gradient resulting in the formation of a ubisemiquinone radical intermediate, which in some conditions is likely to give an electron to O_2_ to form superoxide [16,37]. According to this model, mitochondrial ROS production is necessary and sufficient to induce HIF-1α stabilization, promoting HIF-1-regulated processes, including EMT. This finding is very important for cancer therapy since it identifies the mitochondrial respiratory chain Complex III as a potential target through which EMT, and therefore metastases formation and resistance to therapies, could be inhibited. However, not all Complex III inhibitors inhibit ROS production; as a matter of fact, inhibitors that act at the Qi site are known to increase superoxide anion levels [36,49].

In contrast, and in agreement with other authors [16,37], in our model, the Qo inhibitor Myxo has been shown to inhibit both hypoxia-induced ROS formation and hypoxia-induced HIF-1α stabilization, along with EMT. However, Myxo also inhibits mitochondrial respiration [27], which drastically reduces its potential for successful clinical application. As a matter of fact, the antiproliferative effect observed following Myxo treatment, both in normoxia and hypoxia, could be a confirmation of this last statement. In contrast, Terpestacin (Ter) was shown to bind the UQCRB subunit of complex III and to suppress hypoxia-induced mitochondrial ROS generation without inhibiting mitochondrial respiration and ATP generation [21]. This effect was also confirmed in MDA-MB468 cells.

The effects of UQCRB downregulation on ROS production and HIF-1α and EMT protein levels, evaluated on MDA-MB468 cells in which UQCRB was silenced, namely MDA-MB468/shUQCRB, support the hypothesis that the reduction of the hypoxia-induced ROS increase, through the inhibition of mitochondrial Complex III, can reverse EMT phenotype.

It was perhaps surprising that a comparatively modest, albeit significant, decrease in ROS generation observed in UQCRB-silenced cells should totally prevent HIF-1α stabilization and induce such drastic changes in E-cadherin and vimentin levels as compared to control/scrambled cells. Actually, both complex I and Complex III have been implicated as sources of ROS production during hypoxia [36]. UQCRB silencing selectively prevents ROS generation through Complex III; thus, our observations provide indirect evidence of the role played by Complex III-derived ROS hypoxia in HIF-α stabilization and EMT. Indeed, our results confirm and expand observations reported by several groups regarding the role played by mitochondrial ROS in HIF-1 activation and in the downstream hypoxic response [50,51,52]. Interestingly, a recent article on the same cell line used for this study partially contradicts the role played by HIF-1 in hypoxia-induced changes in some, but not all, EMT markers [42], a discrepancy that can possibly be explained by shorter exposures to hypoxia as compared to the present study and to studies by other groups [53,54,55].

## 5. Conclusions

In conclusion, our results are in agreement with recent data suggesting that the UQCRB subunit of Complex III in the mitochondrial respiratory chain plays a pivotal role in hypoxic signaling, and identifies UQCRB as a potential novel therapeutic target for the development of drugs able to counteract tumor progression due to the EMT, by blocking mitochondrial ROS production [21,23,54]. Interestingly, although the migratory capacity of MDA-MB468 cells was significantly decreased by silencing UQCRB, cell survival was not affected.

A number of authors have reported the effect of antioxidants, such as SkQR1, NAC, Mito-CP, and Mito-TEMPO [41,43,44] on the redox modulation of EMT; however, the involvement of the mitochondrial Complex III UQCRB site in the EMT reversion, due to the inhibition of the hypoxia-induced mitochondrial ROS production and the related decrease in HIF-1α stabilization, represents the novelty of this work and might provide a rationale for the development of drugs able to counteract tumor progression induced by the EMT by blocking mitochondrial ROS production at this site.

Finally, the cell line we have used for this study is a particularly interesting model, being representative of the triple negative adenocarcinoma, an aggressive and untreatable subset of breast cancer, that does not respond to endocrine therapy or other currently available targeted agents [56,57]. Novel therapies addressing this tumor subset are urgently needed, and targeting UQCRB might be an option worth investigating, at least for some forms of these tumor types.

## Figures and Tables

**Table 1:** Percentage of open scratch wound in MDA-MB468, MDA-MB231 and MCF7 cells following 48 h incubation under normoxic (O_2_ 21%) and hypoxic conditions (O_2_ 1%) (mean S.E. of 3 independent experiments; * *p* < 0.05 vs. 0 h; *** *p* < 0.001 vs. 0 h and O_2_ 21%).

	MDA-MB468		MDA-MB231		MCF7	
Time (h)	O_2_ 21%	O_2_ 1%	O_2_ 21%	O_2_ 1%	O_2_ 21%	O_2_ 1%
0	80.0 ± 2.0	75.0 ± 1.0	78.0 ± 2.0	78.0 ± 3.0	76.5 ± 1.5	78.0 ± 3.0
48	54.5 ± 0.5 *	36.5 ± 4.0 ***	59.0 ± 1.5 *	46.5 ± 1.5 ***	74.0 ± 1.0	74.5 ± 2.5

## Data Availability

Data are contained within the article and supplementary material and are available on request.

## Supplementary Materials

The following supporting information can be downloaded at: https://www.mdpi.com/article/10.3390/cimb44110359/s1, Figure S1—Slug and Snail protein levels in MDA-MB468 cell line, following 48 h hypoxia (O_2_ 1%); Figure S2—Effects of MitoTP (100 and 200 mM) treatment and 48 h incubation in normoxia (O_2_ 21%) or hypoxia (O_2_ 1%) on MDA-MB468 viability (mean ± S.E. of 3 independent experiments); Figure S3: N-cadherin, Slug and Snail protein levels in MDA-MB468 cells, following 48 h treatment with Myxo (1 mM) and incubation in hypoxia (O_2_ 1%); Figure S4—Effects of Myxo (1 mM) treatment and 48 h incubation in normoxia (O_2_ 21%) or hypoxia (O_2_ 1%) on MDA-MB468 viability (mean ± S.E. of 3 independent experiments); Figure S5—Effects of Ter (25 mM) treatment and 48 h incubation in normoxia (O_2_ 21%) or hypoxia (O_2_ 1%) on MDA-MB468 viability (mean ± S.E. of 3 independent experiments); Figure S6: Effects of UQCRB silencing on ROS levels in MDA-MB468 cells, following 48 h incubation in normoxia (O_2_ 21%) or hypoxia (O_2_ 1%). Blue line: MDA-MB468/pLVTHM O_2_ 21%; red line: MDA-MB468/scrambledUQCRB O_2_ 21%; purple line: MDA-MB468/shUQCRB O_2_ 21%; light blue line: MDA-MB468/pLVTHM O_2_ 1%; pink line: MDA-MB468/scrambledUQCRB O_2_ 1%; green line: MDA-MB468/shUQCRB O_2_ 1%; Figure S7: Snail protein levels in MDA-MB468/pLVTHM, MDA-MB468/scrambledUQCRB and MDA-MB468/shUQCRB cell lines, following 48 h incubation in normaxia or hypoxia (O_2_ 1%); Figure S8: representative images of the Scratch Wound Healing assay performed in MDA-MB468/pLVTHM, MDA-MB468/scrambledUQCRB and MDA-MB468/shUQCRB cell lines, following 48 h incubation in normoxia (O_2_ 21%) or hypoxia (O_2_ 1%).
