# Supplementary material for: Targeting Mitochondrial ROS Production to Reverse the Epithelial-Mesenchymal Transition in Breast Cancer Cells
Source: Curr Issues Mol Biol. 2022 Oct 29;44(11):5277–93. doi: 10.3390/cimb44110359 (PMC9689492; doi:10.3390/cimb44110359)
Supplement: Supplementary file 1 [file cimb-44-00359-s001.zip › cimb-1991446-supplementary.pdf]

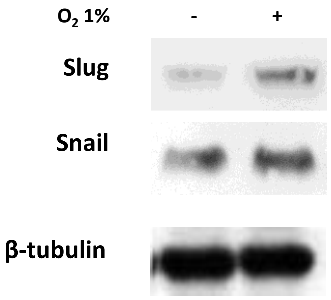

Figure S1 – Slug and Snail protein levels in MDA-MB468 cell line, following 48h hypoxia (O_2 1%).

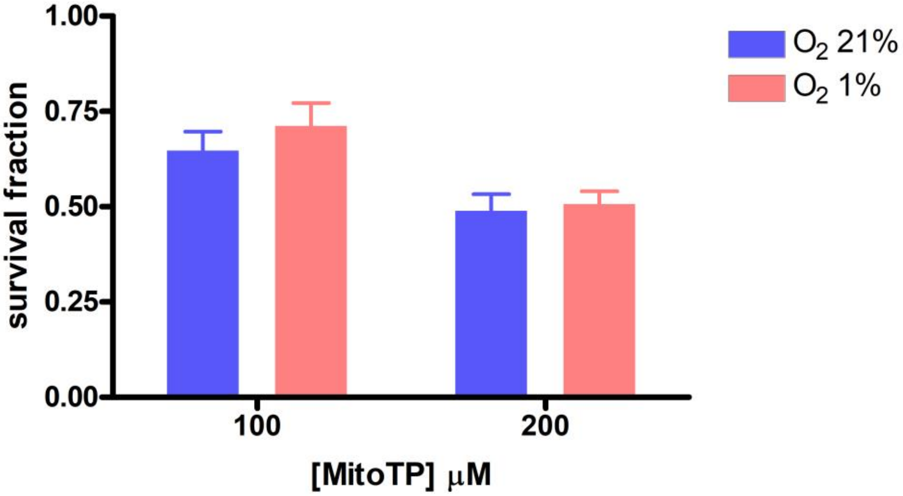

Figure S2 – Effects of MitoTP (100 and 200 μM) treatment and 48h incubation in normoxia (O_2 21%) or hypoxia (O_2 1%) on MDA-MB468 viability (mean±S.E. of 3 independent experiments).

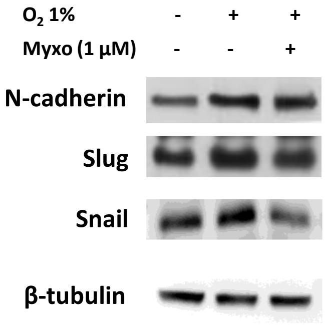

Figure S3: N-cadherin, Slug and Snail protein levels in MDA-MB468 cells, following 48h treatment with Myxo (1 μM) and incubation in hypoxia (O_2 1%).

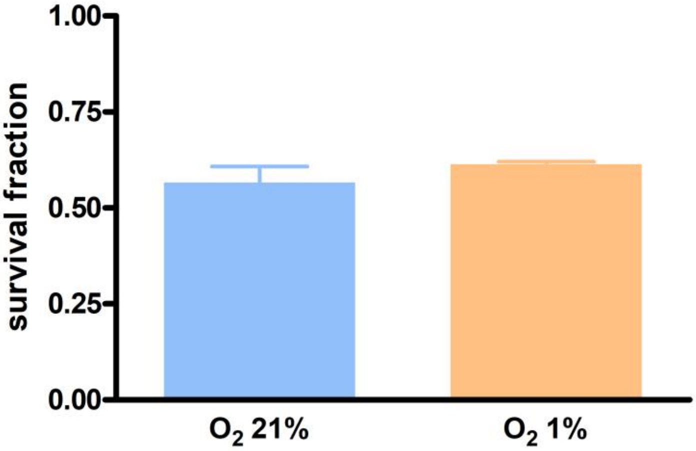

Figure S4 – Effects of Myxo (1 μM) treatment and 48h incubation in normoxia (O_2 21%) or hypoxia (O_2 1%) on MDA-MB468 viability (mean±S.E. of 3 independent experiments).

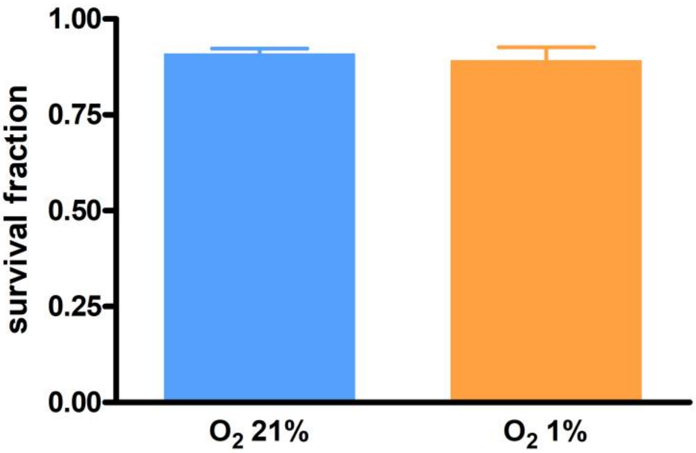

Figure S5 – Effects of Ter (25μM) treatment and 48h incubation in normoxia (O_2 21%) or hypoxia (O_2 1%) on MDA-MB468 viability (mean±S.E. of 3 independent experiments).

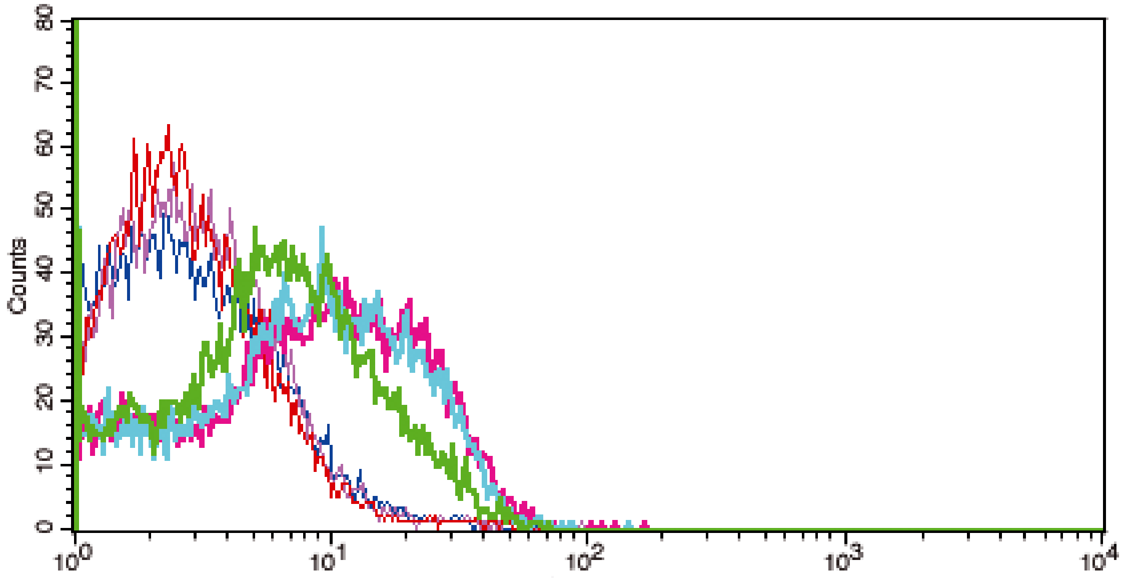

Figure S6: Effects of UQCRB silencing on ROS levels in MDA-MB468 cells, following 48h incubation in normoxia (O_2 21%) or hypoxia (O_2 1%). Blue line: MDA-MB468/pLVTHM O_2 21%; red line: MDA-MB468/scrambledUQCRB O_2 21%; purple line: MDA-MB468/shUQCRB O_2 21%; light blue line: MDA-MB468/pLVTHM O_2 1%; pink line: MDA-MB468/scrambledUQCRB O_2 1%; green line: MDA-MB468/shUQCRB O_2 1%.

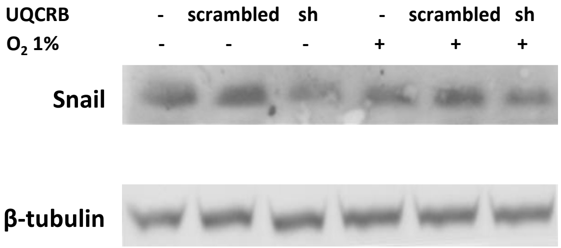

Figure S7: Snail protein levels in MDA-MB468/pLVTHM, MDA-MB468/scrambledUQCRB and MDA-MB468/shUQCRB cell lines, following 48h incubation in normaxia or hypoxia (O_2 1%).

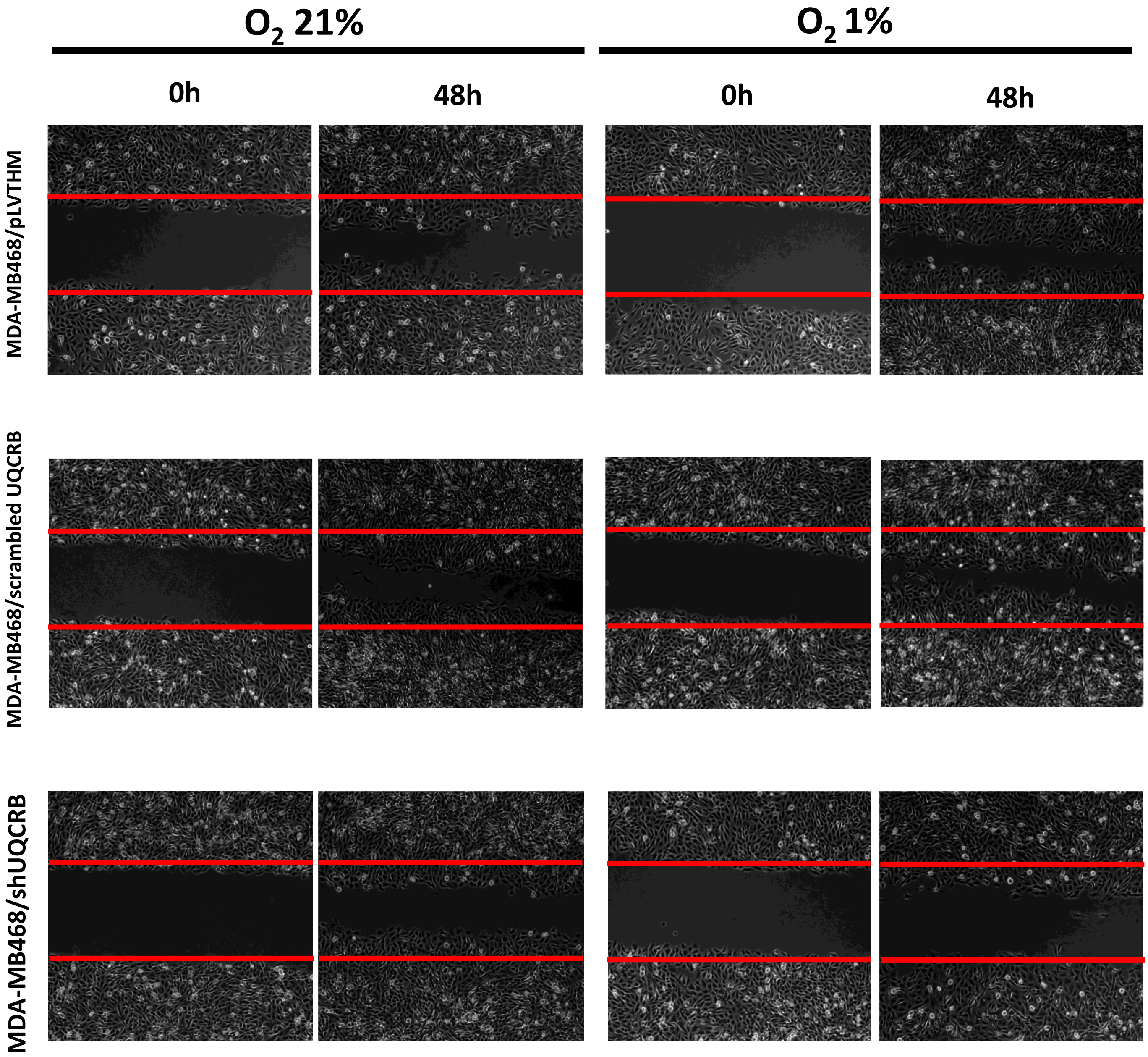

Figure S8: representative images of the Scratch Wound Healing assay performed in MDA-MB468/pLVTHM, MDA-MB468/scrambledUQCRB and MDA-MB468/shUQCRB cell lines, following 48h incubation in normoxia (O_2 21%) or hypoxia (O_2 1%).
